# Orchestrating the fecal microbiota transplantation: Current technological advancements and potential biomedical application

**DOI:** 10.3389/fmedt.2022.961569

**Published:** 2022-09-22

**Authors:** Manisha Nigam, Abhaya Shikhar Panwar, Rahul Kunwar Singh

**Affiliations:** ^1^ Department of Biochemistry, School of Life Sciences, H.N.B. Garhwal University, Srinagar, India; ^2^ Department of Microbiology, School of Life Sciences, H.N.B. Garhwal University, Srinagar, India

**Keywords:** microbiome, dysbiosis, gastrointestinal, safety, extragastrointestinal, gut microbiota

## Abstract

Fecal microbiota transplantation (FMT) has been proved to be an effective treatment for gastrointestinal disorders caused due to microbial disbalance. Nowadays, this approach is being used to treat extragastrointestinal conditions like metabolic and neurological disorders, which are considered to have their provenance in microbial dysbiosis in the intestine. Even though case studies and clinical trials have demonstrated the potential of FMT in treating a variety of ailments, safety and ethical concerns must be answered before the technique is widely used to the community's overall benefit. From this perspective, it is not unexpected that techniques for altering gut microbiota may represent a form of medication whose potential has not yet been thoroughly addressed. This review intends to gather data on recent developments in FMT and its safety, constraints, and ethical considerations.

## Introduction

The fecal microbiota of an individual represents the diversity and composition of their gut microbiota. It is essential for nutrition and metabolism, serves as a protective barrier against pathogenic organisms, and aids in the growth of intestinal epithelium. Individuals’ balanced health is a result of their typical gut microbiota composition, which varies depending on the gender, age, dietary preferences, and lifestyle. Microbial dysbiosis, i.e., microbial imbalance in the human intestine, may lead to the development of several intestinal and extraintestinal disorders such as irritable bowel syndrome (IBS), inflammatory bowel disease (IBD), anxiety, cardiovascular diseases, obesity, type 2 diabetes, depression, and atopy (1). It was hypothesized somewhere during the 20th century that the diseases caused due to significant changes in normal gut microbiota may be treated by restoration of microbial composition (2).

One strategy to restore the normal gut microbiota is orally administering the probiotics containing the desired microbes. Probiotics, in some way, modify the metabolism of the indigenous bacterial flora of the intestine and have a momentary inhabitancy effect on the gut (3). However, the effectiveness of probiotics depends on various factors such as the diversity and functioning of the probiotic cells and the spectrum of their fermentation products (4). Another strategy is administering the solution of desired microbes directly in the intestine. However, in both strategies, the selection of desired microbes is important and needs to be performed carefully (5). Keeping this fact in view, the administration of the bacterial community from the stool of a healthy donor to restore the microbial balance in a diseased person has been a successful technique in the last two decades. The technique is called fecal microbiota transplantation (FMT). Various tools of FMT include colonoscopy, an orogastric tube, enema, or an oral capsule containing the lyophilized organisms (6).

In 2013, FMT was first approved by the US Food and Drug Administration (FDA) for treatment of recurrent *Clostridium difficile* infection (rCDI). Afterward, the technique has emerged as a treatment for a myriad of gastrointestinal and also for non-gastrointestinal disorders, although little is revealed about its mode of action or long-term side effects. Interestingly, there are varying pieces of evidence to substantiate its use. Since this new paradigm implies that many diseases are caused, at least in part, by microbiota dysfunction, it urges more research into FMT as a treatment for various disorders. Emerging research into the gut microbiota, which play crucial roles in immunity and cell metabolism, has sparked interest in this therapeutic intervention. Moreover, with the rising number of FMT treatments and clinical studies, there is an imperative need for standardized regulations to ensure the safety of patients and the targeted improvement of safe and sustainable, rationally designed microbiota-based medicines.

The purpose of the current article is to gather the most recent information on FMT, its challenges, and future prospects. This review will concentrate on the rationale and challenges of using FMT against human diseases and regulatory and ethical concerns. Information has been gathered from the pertinent literature of various databases published mostly over the last 10 years. The field of microbiota-related disorders is still in its early phases; therefore, there is a necessity for further investigation into FMT for its effectiveness, risk profile, and long-term implications.

## FMT for intestinal disorders

In the last decade, FMT has been proven as a successful treatment tool for recurrent CDI with an effective rate of 90% (7). Consequently, the technique has been recognized in several standard guidelines for treatment of CDI including those of the WHO and US FDA. More notably, scientists are now focusing on clinical trials of FMT for treatment of IBD and ulcerative colitis (UC). The initial data of these clinical trials are suggestive of the significant therapeutic role of FMT in both IBD and UC (8, 9). However, the technique was not as effective as in the case of CDI. To improve the effectiveness, modifications of FMT such as step-up or intensive dosing multisession FMT were employed in the case of IBD and UC patients (9, 10). Nevertheless, the data that are currently available show that FMT is a more successful method of treating these intestinal conditions than administering antibiotics (11).

Sood et al. conducted a study among 61 UC patients in clinical remission. Participants were randomly assigned to receive either FMT or placebo (12). Maintenance of clinical remission at 48 weeks was achieved in 87.1% of patients receiving FMT compared with 66.7% receiving placebo. There was a statistically significant impact of FMT on endoscopic remission (FMT: 58.1% compared with placebo: 26.7%, *p* = 0.026) and on histological remission (FMT: 45.2% compared with placebo: 16.7%, *p* = 0.033). It is indicated that FMT in UC patients could help sustain endoscopic, histological, and clinical remission (6).

## FMT for extraintestinal disorders

In addition to gastrointestinal diseases, the use of FMT has been extended to several extragastrointestinal ailments in the recent past. Numerous investigations have evaluated the hypothesis of changing the gut microbiota as a promising treatment for metabolic diseases like obesity and other metabolic syndromes (13). Recently, the efficacy of FMT has been observed in changing not only the gut microbiome composition but also the host metabolome and epigenome of immune cells (14). Several case reports and animal models have also revealed the probable therapeutic effects of FMT in patients with severe multiple sclerosis (15), autism (16), multidrug-resistant (MDR) infections (17), and multiple organ dysfunction in critical patients (18). Furthermore, the studies also demonstrated the positive effects of immunotherapy on melanoma with FMT in an animal model and clinical trial (19, 20).

### Metabolic disorders

Numerous evidence-based studies demonstrate the significance of gut microbiota in a range of metabolic ailments (21, 22). Changes in gut microbiota composition have also been reported in obese humans with a shift in the ratio of *Firmicutes* and *Bacteroidetes* and the presence of *Lactobacillus* spp. (23). Recent research studies have shown that the *Clostridia* strains secreting butyrate were found in fewer numbers in the intestine of patients with type 2 diabetes mellitus, whereas non-butyrate-producing *Clostridiales* were found in a higher number of studies that showed that both insulin sensitivity and levels of butyrate-producing intestinal microbiota were markedly increased after microbiota transplantation (24).

FMT has recently been shown to be helpful for regulating blood glucose and blood pressure in type 2 diabetic patients when supplemented with a particular diet over the course of a 3-month investigation. After receiving therapy, these patients’ gut microbiota's 16S rRNA sequence homology analysis showed the abundance of *Bifidobacterium*, while *Bilophila* and *Desulfovibrio* had significantly decreased. Additionally, a fall in blood pressure, blood lipids, blood glucose, and body mass index was favorably correlated with the presence of *Bifidobacterium*. (25).

### Neurological disorders

Studies on germ-free mice have suggested the concept of bidirectional brain–gut–microbiota axis. The imbalance in this axis leads to various neurological disorders such as anxiety, depression, Alzheimer's dementia, and Parkinson's disease. (26, 27). These studies also suggest that restoration of the balanced gut microbiota may improve the health conditions of patients suffering from brain disorders. However, the efficacy of FMT in treating these ailments is not widely explored clinically. A few recent case studies of FMT in human subjects with neurological disorders are mentioned below.

Recently, a 90-year-old woman with severe CDI and Alzheimer's dementia was treated with FMT by Park et al. (28) at Inho University Hospital in Incheon, South Korea. The comparative analysis of results before and after the transplantation revealed significant changes in her fecal microbiota composition and considerable improvement in her cognitive functions. The research also confirmed that there is a significant link between gut bacteria and cognitive functioning.

Another clinical investigation was conducted by Segal et al. (29) at Soroka University Medical Centre, Israel, on six patients (three males; three females; age 60 ± 13 years) suffering from Parkinson's disease and constipation. After 4 weeks following transplantation, the results indicated a considerable improvement in the motor, nonmotor, and constipation scores of five patients, which persisted. The modifications in these scores after 6 months of transplantation ranged from −13 to 7 points for motor scores, from −2 to −45 points for nonmotor scores, and from −12 to 1 point for constipation scores.

Doll et al. (30) transplanted the fecal microbiota in two major depressive disorder (MDD) patients as add-on therapy for the first time. Both the patients experienced a reduction in the severity of their depression symptoms after 4 weeks of the transplantation. The study recommended the further trial of FMT for treatment of MDD.

## FMT vs. other approaches for reasserting the healthy gut flora

Microbiota intervention techniques can be accomplished by utilizing a multitude of approaches, including diet, prebiotics, probiotics, postbiotics, antibiotics, phage therapy, bacterial consortium transplantation, and FMT. The use of microbiota modification to promote health is emerging as a potent therapeutic strategy against a plethora of diseases. Numerous host factors, such as genetics, metabolism, exposure to the environment, microbial composition, and activity, may influence these interactions; yet, the intricacy of microbiota–host crosstalk is still not fully understood. As we learn more about the interactions between the microbiota and the host, we will be able to pinpoint potential targets and address a number of unresolved issues, including the timing of treatment, host aging, the right approach, the best strain, individualized prevention or therapy, and the use of “live” or “dead” microorganisms.

Probiotics approach is typically seen to be secure and well-tolerated in healthy individuals; however, their safety profile has been questioned in patients with pre-existing ailments. One of the major concerns is probiotic translocation, which is the term for the introduction of live bacteria into extraintestinal locations and the subsequent systemic or localized infections. The probable spread of antibiotic-resistant genes by horizontal gene transfer, which refers to the dispersal of mobile genetic materials within and across species, poses another concern associated with long-term probiotic use. However, research on the resistance gene transfer caused by probiotics is still mostly confined to preclinical settings, and many challenges about its implications and therapeutic relevance remain unanswered. Due to numerous confounding variables in clinical situations, it has been very difficult to establish a linkage between probiotic consumption and the development of resistance. Along with this, other issues to consider when utilizing probiotics include systemic infections, harmful metabolic processes, and excessive immunological stimulation in vulnerable people (31). Similarly, several factors must be taken into account when drawing inferences about the impact of using prebiotics, which are substances that host microbes utilize in a particular way to confer a health benefit. It is claimed that prebiotic interventions may have various effects on different people and, even more startlingly, may occasionally have negative consequences on the host, depending on the somatic and genetic background (32). Another approach is the use of postbiotics, which are the soluble byproducts and metabolites released by the gut microbiota having biological effects on the host. For some probiotic strains, the desired effect is not produced by living bacteria but rather by the conditioned medium (or culture supernatants). Therefore, compared to ingesting living microbes, postbiotics may occasionally be a successful yet safer technique (33). Postbiotics research is an emerging but still mostly unexplored field. It has been extremely difficult for researchers to identify the molecules responsible for the therapeutic action because of the enormous number and diversity of metabolites that have been produced; therefore, to describe the safety profile of a particular molecule in preclinical and clinical settings is cumbersome.

The probiotics principle is largely followed in the modification of the gut microbiota by FMT; however, rather than administering a single strain to the patient, a community of microbes is employed in this procedure. FMT appears to provide a myriad of benefits over other modulation techniques. With its long-term engraftment, it can be structured as a single-dose regimen, conferring therapeutic benefits over probiotics and prebiotics, whose colonization appears to be transitory, while also boosting microbial diversity and not disrupting microbial gut ecology as in antibiotic treatment, as the latter also reduce the overall diversity of beneficial bacteria in the gut (32).

The therapeutic effects of FMT can be attributed to an expanded variety of bacteria, viruses, fungi, and archaea that can engraft into the recipient host and enhance the functional diversity of the gut microbiota. FMT is also being tested in nearly 300 clinical trials for various disease indications, including autoimmune diseases, neurological issues, cancer, graft vs. host disease, and metabolic and gastrointestinal disorders. Intriguingly, FMT may be deployed in unusual circumstances that would justify its application, such as when a patient cannot receive antibiotics due to severe sickness, in case of antibiotics intolerance, or when a patient is severely unstable to undergo surgery (34). Furthermore, the quick development and widespread interest in FMT are not just due to its effectiveness as a treatment for rCDI as described above. The abundance of “biological material,” the popular interest in alternative and natural therapies, and the possibility of self-administration are further factors (35).

## Challenges in fecal microbial transplantation

FMT has emerged as an important and efficacious therapeutic strategy for restoring intestinal microbial balance and other ailments. However, it faces a variety of challenges and barriers in being adopted as an intervention for the same. The major challenges and issues in this concern are as follows.

### Donor selection

Multiple studies suggest that donor selection is a fundamental challenge in implementing the FMT program globally (36). FMT needs a stool donor that must be nonobese and present in overall good health with no risk factor of infectious diseases or any other chronic diseases with no objection in frequent donation. Although the criteria and conditions for donor selection seem simple and not that selective, finding a sufficient number of donors to meet the needs of FMT is not always an easy task. To get an ideal fecal sample for FMT, along with an intuitive stool assessment, the donor must undergo serologic and hematological screening with medical, family, and personal history assessment to prevent the transfer of communicable diseases from the donor to the recipient. Donor selection is a challenging and difficult task as the gut microbiota is a complex entity, making screening procedures expensive, constrained, and contentious (37, 38). Along with the screening procedures, there are concerns regarding the donor profile, such as whether or not children, pregnant women, or nursing mothers should be prohibited from donating and whether or not the donor's dietary history should be taken into account (39). It has also been confirmed from the data of large stool banks that a high rate of donor drops out due to the high commitment required from donors, and physicians often abandon FMT due to the complexity and cost involved in the process (40). The assessment studies of microbial colonization in recipient's gut after successful FMT revealed that the rate of colonization of new donor-derived strains is less in comparison to the strains that were already existing in the recipient, hence shifting the focus from donor selection to proper donor–recipient matching (38).

The lack of clear evidence-based guidelines and varying recommendations among medical societies also generate challenges in donor selection. A significant portion of studies published on FMT does not explicitly confirm the criteria for donor selection, risk factors, and pathogens to be screened, as well as the timing and frequency of clinical investigations (41). It is a subject of debate to date whether preference should be given to family-related donors (patient-identified donors) or unrelated donors. Using a patient-identified donor for FMT may uphold confidentiality among the patient and donor, but in an emergency situation when prompt therapy is needed, finding and screening such donors are not feasible due to time constraints (39). The facility of a stool bank has resolved this issue where fecal material could be stored, which provides fast and safe access to donor feces and supports the hospitals for FMT. However, some recent studies disclosed that there are no advantages in preferring related healthy donor volunteers over unrelated healthy donors (42, 43, 38). Other challenges associated with FMT donor selection are the age limit of the donor, which is not specified, and lack proper guidelines. Furthermore, the gut microbiota is known to be influenced by dietary intake, and its implications for FMT donor selection are yet to be investigated (44). The concept of many donors for a single patient through pooling donor stool in terms of maintaining high diversity inocula needs further standardization and investigation.

### Sample handling

Investigations are being conducted to establish the most effective method for preparation and administration of FMT. The outcomes of FMT are greatly influenced by the sample handling from collection to administration. Variations in the procedures of stool mixing, fecal sample concentration, and the volume of the sample being transplanted significantly affect the patient response and therapeutic outcomes (45). Generally, the stool samples are processed by making suspension in isotonic solution followed by filtration and subsequently either transplanted or cryopreserved. Majority of fecal microbiota are anaerobic; therefore, stool samples should be processed within 6 h of defecation so that microbial viability can be maintained, although consensus is lacking among guidelines regarding anaerobic processing of fecal material (46). Despite a few worldwide existing stool banks, there is a lack of standard protocols for producing FMT material, and because of varying factors among the institutions providing FMT therapy, a condition of uncertainty persists (47). Utilization of frozen samples in place of fresh fecal samples still remains a matter of investigation among research communities in terms of its efficacy, scientific virtues, and establishing practicality of FMT in the clinical context. The inherent constraints in preparing fresh fecal matter, such as the selection of acceptable donors and frequent screening, as well as the case-by-case processing of samples, make the process time-consuming, expensive, and unpredictable (45). In a randomized clinical trial, a single treatment of fresh, frozen, and lyophilized microbiota was given *via* colonoscopy to patients suffering from rCDI. Remission of CDI was achieved 100% with patients who received fresh FMT, 83% with patients who received frozen FMT, whereas it was 78% with patients who received lyophilized FMT (48). Utilization of lyophilized preparations for FMT has advantages in that deep freezing is not needed and lyophilized preparations can be potentially encapsulated. Although lyophilization is used for decades, there is still a scope for optimizing the protocols for preserving essential taxa present in the complete fecal microbiota (49). It has been noticed that there is a lack of longitudinal studies demonstrating the optimal freezing time before microbial function declines, and more investigations are needed to describe statistical significance between fresh or frozen samples so that a standardized and effective methodology of stool sample preparation can be developed. Furthermore, the use of biocides, such as ethanol, is very common to maintain pathogen-free inocula. However, there are certain biocide-resistant microorganisms that may not be sufficiently inactivated and increase the risk factor (50). Additionally, the use of ethanol may also affect and eliminate the healthy commensal strains that have huge clinical significance and play a key role in microbiome restoration.

### Modes of administration

The appropriate dose, frequency of administration, and route of administration are additional obstacles to broader utilization of FMT. The recommended amount of stool is ≥50 g for FMT, but the ideal method of processing and the size and frequency of the FMT dose need standardization (51). Methods for FMT implementation can be grouped into four categories (52):

1. Focus on the upper digestive tract, carried out by gastroscopy or nasal cavity tube.
2. Focus on the lower intestine, carried out by colonoscopy, retention enema, and colonic tube.
3. A combined approach.
4. The oral capsules.

Each mode of administration targets different areas of the small and large intestine to deliver inoculum and bypass certain parts; for example, rectal enemas can target the rectum and left colon, colonoscopy can cover the entire colon, and oral ingestion deliver to the proximal and mid small bowel (45). Patient's condition and willingness must be considered before implementing any FMT approach. Comparing the upper and lower routes of administration, the lower route was found to be proficient, whereas the upper routes of administration were associated with slight adverse effects; however, there is a lack of head**-**to**-**head clinical trials to evaluate the effect of route of administration (53). Gundacker et al. (54) also reported that upper delivery routes do not result in a cure as often as lower endoscopy. Although upper delivery routes may be beneficial in the case of mild disease patients, severely diseased patients need FMT by lower endoscopy. It is still unclear to select the best way to perform FMT due to the scarcity of related comparative studies (52). In addition, uncertainty persists in figuring out whether a combination of two administration routes is superior to a single administration route or not. The mode of administration is associated with some adverse events, which are rare but should be considered. Some of them include endoscopy-mediated complications such as perforation and bleeding and sedation-coupled side effects (55). Avoiding administration-related adverse effects, oral capsules for FMT have gained more attention due to their convenience and efficacy (46). Compared to colonoscopy, oral capsules are less resource-intensive and noninvasive and, therefore, have arisen as an attractive option in recent years. The use of colonic release capsules, which target the site of the disease, increased credibility in FMT. The oral route is the most convenient and preferred route for colon-specific drug delivery, but other routes may be used. Oral FMT capsules improved both access and acceptance among patients. Nevertheless, the oral bacterial capsule approach is suitable for the narrow patient population who do not have esophageal, dysphagia, or pharyngeal aspiration issues and can swallow 20–40 capsules (56). Recently, Kao et al. (57), in their comparative investigation, established that capsules for colonoscopic delivery were equally efficacious, although more clinical trials needed to be evaluated.

### Colonization resistance

The gastrointestinal tract is a hub of a range of microbial communities involved in crucial functions of the host. The development and functions of the innate and adaptive immune systems are greatly influenced by the intestinal microbiota. Healthy microbial communities provide a protective role for the host against invading bacterial pathogens through a mechanism called colonization resistance (58). Any alteration or imbalance in the community structure of these healthy microbiota leads to the loss of colonization resistance ability and subsequently enhances the chances of pathogen colonization (59). The disruption of the gut microbiota's equilibrium, the loss in colonization resistance, and the rise in intestinal colonization by antibiotic-resistant organisms are primarily caused due to the frequent and inappropriate use of antibiotics (58, 60). Inappropriate use of antibiotics also leads to the expansion of MDR microorganisms in the gut. FMT is increasingly being used to eliminate MDR bacteria and to restore colonization resistance (61–63). Since the discovery and evaluation of the microbiota-derived bacterial population and their immunomodulatory compounds are still in the early stages, the reliability of FMT is still under question (64). Moreover, gut microbiota composition varies from person to person, and choosing FMT is as close to a “one-size-fits-all” approach as possible. Although there are several existing hypotheses to elaborate on how inoculation of whole stool contents from healthy donors prevent ailments, we have insufficient mechanistic understanding in this concern.

## Safety concerns and ethical issues with FMT

Along with the issues like accessibility, acceptability, lack of standardization, and regulatory complexity, FMT is also linked with safety concerns. The US FDA considered fecal microbiota as a biological product and a new investigational drug (65). Although the clinical resolution of CDI through FMT was reported up to 92% (66), being effective and efficacious for CDI, FMT still remains an investigational treatment (67, 68). In most clinical trials, FMT is considered a safe method; still, there is a scope to make it trustworthy among the population. There are several mild to moderate short-term adverse effects associated with it. Symptoms of abdominal cramping and bloating, mild diarrhea, nausea, fatigue, and headaches are some of the consequences that can be noticed upon FMT administration (38). The majority of the patients get relief from these adverse effects within a couple of hours of treatment, whereas prolong symptoms persist in some patients. After a few weeks of treatment, complications like blood in the stool and infections in the urinary and respiratory systems were noticed in some patients, even though these symptoms were unrelated to the therapy. (45). Despite these observations, FMT appears to have negligible short-term side effects. However, there is insufficient research on its long-term effects, particularly on recolonization, the transmission of resistance genes, and the colonization of new bacterial strains (69). Thus, long-term safety studies involving large populations are unquestionably required to rule out the unidentified risk of changing microbiota. An FMT National Registry was recently established by the American Gastroenterology Association with funding from the National Institutes of Health (NIH). Its goal is to enroll 75 centers and track 4,000 FMT patients over 10 years to have long-term follow-up and clearly articulate the long-term safety profile (46).

Most of the studies reported no serious adverse reaction to FMT, whereas some undesirable consequences were noticed after the alteration of gut microbiota in few cases. Some of them were obesity, diabetes, asthma, autism, IBD, and immunological diseases such as peripheral neuropathy, Sjogren's disease, idiopathic thrombocytopenic purpura, rheumatoid arthritis, and fever (38, 58, 70–72). However, in most of these cases, it was unclear whether these adverse events were coincidental or resulted due to FMT. Moreover, in a certain group of patients such as immunosuppressed individuals, doubt regarding the safety of FMT still persists. Concerns about the possibility of post-FMT infection in immunocompromised patients have been raised.

A rare case has been noticed that involves the death of a patient due to the acquisition of an extended-spectrum beta-lactamase (ESBL)-producing *Escherichia coli* from donated stool (73). This occurred due to the nonimplementation of ESBL screening of donor stool. Following this case, FDA responded by posting a national alert on its website and mandating additional screening for ESBL (74). Due to this incidence, it is clear that FMT may be associated with unidentified bacteria that may provide certain risk factors. As a preventive measure, screening and selection procedures should be continuously examined, and vigilance over potentially new pathogens is necessary. Additionally, it has been submitted that mental health is greatly influenced by gut microbiota (75). In this context, the issue of whether FMT is to blame for a change in neurological condition or linked to the possibilities of carrying along neurological and mood issues that can impact a person's personality is raised. Therefore, there are ethical implications regarding informed consent and FMT choice, and more research is required to address these concerns. FMT has long been associated with safety issues, yet there are still important gaps in our comprehension of its effectiveness and safety. However, unless obligatory standards are developed with the FDA review and approval, these hazards will still exist since there are currently no mandated guidelines for their execution (50). The generalizability of the results of FMT studies was constrained by the use of nonstandardized treatment regimens, the lack of control groups, and the prolonged follow-up periods. International stool banks were established to address these problems and restrictions and to improve the accessibility of FMT. These banks follow strict safety protocols to prevent treatment-related adverse serious events so that the risk associated with FMT from thoroughly vetted donors can be avoided (76). However, the risks persist because donor samples are usually subjected to screening of common transmissible pathogens, while unknown transmissible pathogens or commensals that can impact the recipient physiology in the short or long run may be overlooked (77). In the context of the recent SARS-CoV-2 outbreak, stringent policies are required for the screening of FMT donors to ensure patient safety. In this concern, FDA directed that further safety measures are required for any investigational use of FMT, whether under an Investigational New Drug Application (IND) on file with the FDA or under FDA's enforcement discretion policy (78).

FMT is associated with some real and theoretical risks that raise a number of ethical concerns when it is offered to a patient, a few of which are listed below.

### Informed consent

It is necessary to have informed voluntary consent on the part of patients to carry out research and clinical trials. An informed consent typically involves three elements: the capacity to consent, voluntariness, and information. In FMT, insufficient information rather than a lack of the capacity to consent is the primary cause of issues (79, 80). Informed consent may be difficult to obtain due to the lack of information about the possible adverse effects, short- and long-term impacts of gut microbiota perturbations on systemic health, and the untested nature of the treatment. (81). It is important to disclose all the FMT-associated risk factors at the time of the informed consent process, and FMT practitioners must discuss the preclinical research and long-term risk of FMT. Quite often, clinicians who are not familiar with the preclinical studies may inadequately appreciate the risk of FMT and their patients as well. FMT is a rapidly evolving field where clinical experience and discrete understanding of therapeutic mechanisms are not in parallel run; therefore, clinicians face difficulties in educating patients about unresolved concepts associated with FMT (82). Although FMT seems natural, safe, and possibly economical, the investigators always have concerns about the lasting effect of FMT on recipients. The FDA has not yet approved FMT for any medical indication except CDI, for which FDA has allowed clinicians to exercise enforcement discretion in case of patients not responding to standard treatment (80). In its current policy regarding FMT-based treatment of CDI, the FDA specified that the clinician should include a statement that the use of FMT for CDI cure is investigational and mention the discussion of associated risks in the informed consent (82). Ma et al. (81), in their article, demonstrated that “patient's autonomy may be compromised by their stress and desperation, and consequently affect their ability to give informed consent.” Bunnik et al. (80) contended against this concept and concluded that, unless demonstrated otherwise, patients are typically presumed to be capable of providing informed consent.

### Suitable healthy donors

Although it seems that the raw material for FMT can be easily available, searching for a healthy donor is not easy. Immunological compatibility between the donor and recipient is not necessary for FMT, although meticulous screening of the donor is necessary to prevent catastrophic complications. The efficacy of FMT greatly depends on a healthy donor. Defining and identifying a suitable healthy donor is a prime step to make FMT successful, which poses certain ethical issues. In view of current limited knowledge about the composition of gut microbiota profile, it is difficult to define an optimal donor for a patient to target a disease. Selection of healthy donors appears simple, but the present strict and evolving screening standards for donor selection reduce the availability of donor count (39). A range of donor screening protocols are available and being implemented globally, but there are inconsistencies between these protocols (39, 83, 84). Issues like confidentiality, protection, and anonymity may come up throughout the donor selection process, so it is essential to understand how to deal with these issues.

### Commercialization and potential exploitation of vulnerable patients

The commercial use of FMT put forward major ethical issues. There are many websites involved in advertising the home DIY (Do-It-Yourself) FMT kits as direct-to-consumer products (85). Commercial providers can sell these DIY FMT kits directly to the patient, which may promote self-administered transplantation. Skipping the essential need for standard medical care and the guidance of certified healthcare professionals, patients may put their safety at risk. Moreover, if self-administered transplantation produces unfavorable results, the patient would lose faith in FMT, regard it as a dubious medical practice, and condemn FMT on social media platforms (81). Advertising FMT therapies that exclude the requirement of health advisors may promote the chance of patient exploitation rather than their empowerment. Providing the FMT for commercial purposes can make regulatory control more difficult because it may create vagueness among therapy and cosmetic treatment. Therefore, FMT should only be performed by certified healthcare professionals who are competent in providing appropriate informed consent processes and counseling services to uphold quality standards and assure the patient's safety.

### Public health implications

The literature largely ignores the ethical and social implications of FMT for public health. According to Ma et al. (81), it can be demonstrated in two ways: first, it would be preferable to utilize FMT early rather than as a last resort to treat a condition, notably rCDI (where antibiotic treatment is not that successful), to avoid the usage of antibiotics. This will support the public health goals by reducing antibiotic resistance issues. Second, owing to the shared nature of human gut microbiomes (shared by horizontal and vertical transfer) across family and community, any modulation in an individual's gut microbiome may lead to unknown health outcomes for that individual's family and community having overactive (those with ulcerative colitis) or suppressed (transplant recipients) immune systems. These unknown outcomes may be either harmful or beneficial or there may be no effect at all, but it is to be noted that an individual's health may be directly affected by the health choices of others. As far as bioethics is concerned, there are implications for autonomy since individual microbiomes may be altered against their will or ignorantly that are inimical to them (86). From a public health perspective, the collective microbiome of a population will change if the major group of a population changes their microbiome, and this may cause safety implications (86).

## Future of fecal microbiota transplantation

The Human Microbiome Project (87) and the European-based Metagenomics of the Human Intestinal Tract (MetaHIT) consortia, which were established to explore the human gastrointestinal microbiome, have made significant progress in our understanding of the gastrointestinal microbiota (GiMb) in recent years. GiMbs are no longer speculated only as harmless colonizers of the intestine but as active participants attributing to human health and immune-mediated diseases.

Interestingly, it is worth noting that the structure and function of bacterial communities have been the focus of most research on the gut microbiome to date, although it includes archaea, viruses, and eukarya as well (88). On the other hand, the research on the contribution of viruses including bacteriophages, fungi, and protozoa on the impact of the gut microbiome is still limited (89), which, if explored, may revolutionize the usage of FMT and its prospective therapeutic approaches. FMT could eventually be replaced by defined consortia of bacterial or single strains that have been deliberately selected based on their mode of action in the future. This is being worked on by many business and noncommercial groups and organizations (90). For instance, asthma frequently starts in childhood when gut microbiota is still evolving (91). Emerging evidence has demonstrated a link between dysbiosis of the gut microbiota and asthma (92). Interestingly, it is still a relatively new research area; evidence to date implies that the gut microbiota might become a rich target for allergic asthma prevention or control. Probiotics, fecal microbiota transplants, and bacterial lysates have not yet entered clinical practice as a means of preventing and treating microbiome dysbiosis and restoring a healthy microbiome (93). As a result, further mechanistic research is needed to understand better the role of microbial composition in asthma etiology, and FMT could be a prospective treatment option for asthma.

It is imperative to note that until recently, the majority of knowledge about gut microbiota was gathered *via* labor-intensive, low-throughput culture-based approaches. Furthermore, they require certain conditions for bacterial cultivation (an anaerobic environment), which means that a substantial portion of the gut microbiota is excluded. Investigators now have the tools and capabilities to investigate gut microbiota using culture-independent methods owing to the introduction and subsequent implementation of metagenomics and next-generation sequencing technologies. Such developments will undoubtedly revolutionize the FMT technique by addressing a number of unanswered problems.

In addition, the future of the FMT depends on a plethora of other factors, including the identification of conditions for which microbiota-based medicines have true therapeutic implications, well-designed clinical trials of FMT to validate the role of microbiota in disease, and the identification of specific microbial metabolites responsible for such impact. In some situations, a customized or even personalized approach could provide patients with the best and safest treatment interventions.

The use of standardized, highly specialized laboratories for stool preparation is required to incorporate adequate screening procedures for GiMb to safely deliver FMT to patients. Expertise in the recording and maintenance of donor's health and lifestyle variables; sample collection, preparation, and storage protocols; rigorous screening of donor material; and standardized mechanisms for reporting adverse events are all required in these laboratories. As a result, such facilities and operations have the potential to revolutionize the large-scale application of the FMT in the future.

FMT has always been linked with a risk for some patients owing to the complexity and heterogeneity of donor feces and our poor understanding of the ecological dynamics that shape the microbiota. Next-generation microbiota-based therapies will likely become the preferred approach due to such issues. Rigid clinical trials are more suited to these defined interventions using rationally selected combinations of microorganisms or their products. These therapies will allow for the determination of optimal treatment regimens and the development of risk levels that can be managed more reliably than FMT. At the same time, as we broaden FMT indications and its use grows more widespread, we must stay attentive to any long-term safety risks that may occur from GiMb modification.

## Conclusion

FMT has now been accepted by mainstream clinicians as a legitimate therapeutic alternative in the last several years as an ingeniously simple and cost-effective therapy. FMT looks to be a reasonably safe treatment, with the majority of adverse effects being modest and self-limiting, according to short-term follow-up. The long-term consequences of FMT are unknown, and future research should focus on this. FMT's efficacy in *C. difficile* infection is undeniable. However, more randomized controlled trials’ pieces of evidence are needed before wide\-scale adoption of FMT as a therapeutic benefit beyond recurrent CDI. It is being researched as a treatment for IBD, IBS, and metabolic syndrome/insulin resistance, among other disorders. It is envisaged that FMT's indications will extend, and it will become more widely available and accessible as we get more results from the clinical trials. Incidental evidence of FMT efficacy in comorbidities could broaden the scope of FMT indications to include illnesses not previously associated with gastrointestinal dysbiosis and for which therapeutic GiMb modification could be useful.

It should be emphasized, however, that fecal microbiota is a complex starting matter, and those intending to reverse-engineer it will most probably have to figure out the mutual interactions of the microbial communities in the samples and its mechanism of action. Considering this, it may be a long time before an appropriate blend of microorganisms is established and eventually implemented in the therapy. Until that time arrives, medical researchers should concentrate on making FMT as safe and efficacious as possible by following consensus advice suggestions and funding interventional FMT studies.

## Data Availability

The original contributions presented in the study are included in the article/Supplementary Material; further inquiries can be directed to the corresponding authors.
